# *RP-REP *Ribosomal Profiling Reports: an open-source cloud-enabled framework for reproducible ribosomal profiling data processing, analysis, and result reporting

**DOI:** 10.12688/f1000research.40668.1

**Published:** 2021-02-24

**Authors:** Travis L. Jensen, William F. Hooper, Sami R. Cherikh, Johannes B. Goll

**Affiliations:** 1 The Emmes Company, 401 North Washington Street, Suite 700, Rockville, MD 20850, USA

**Keywords:** RP-REP, ribosomal profiling, RNA-Seq, transcriptomics, differential gene translation, pathway enrichment, translational efficiency, reproducible research, cloud computing, AMI

## Abstract

Ribosomal profiling is an emerging experimental technology to measure protein synthesis by sequencing short mRNA fragments undergoing translation in ribosomes. Applied on the genome wide scale, this is a powerful tool to profile global protein synthesis within cell populations of interest. Such information can be utilized for biomarker discovery and detection of treatment-responsive genes. However, analysis of ribosomal profiling data requires careful preprocessing to reduce the impact of artifacts and dedicated statistical methods for visualizing and modeling the high-dimensional discrete read count data. Here we present Ribosomal Profiling Reports (RP-REP), a new open-source cloud-enabled software that allows users to execute start-to-end gene-level ribosomal profiling and RNA-Seq analysis on a pre-configured Amazon Virtual Machine Image (AMI) hosted on AWS or on the user’s own Ubuntu Linux server. The software works with FASTQ files stored locally, on AWS S3, or at the Sequence Read Archive (SRA). RP-REP automatically executes a series of customizable steps including filtering of contaminant RNA, enrichment of true ribosomal footprints, reference alignment and gene translation quantification, gene body coverage, CRAM compression, reference alignment QC, data normalization, multivariate data visualization, identification of differentially translated genes, and generation of heatmaps, co-translated gene clusters, enriched pathways, and other custom visualizations. RP-REP provides functionality to contrast RNA-SEQ and ribosomal profiling results, and calculates translational efficiency per gene. The software outputs a PDF report and publication-ready table and figure files. As a use case, we provide RP-REP results for a dengue virus study that tested cytosol and endoplasmic reticulum cellular fractions of human Huh7 cells pre-infection and at 6 h, 12 h, 24 h, and 40 h post-infection. Case study results, Ubuntu installation scripts, and the most recent RP-REP source code are accessible at GitHub. The cloud-ready AMI is available at AWS (AMI ID: RPREP RSEQREP (Ribosome Profiling and RNA-Seq Reports) v2.1 (ami-00b92f52d763145d3)).

## Introduction

While the principles for ribosomal profiling (RP) were invented decades ago, the application of next-generation sequencing recently set the stage for genome-wide assessments of translation at codon resolution ^1– 3^. The technique makes use of the facts that mRNAs that undergo translation in ribosomes can be fixated to each other using certain chemicals and that the joint ribosome/mRNA complexes can be isolated using chromatography after mRNAs not protected by ribosomes have been degraded using ribonucleases. Following isolation, as for RNA-Seq, mRNA fragments are reverse transcribed and sequenced. The resulting reads may not only represent true ribosomal footprints (reads that originated from mRNA bound to a ribosome, typically ranging between 25 to 35 nt in length) but artifacts/contaminants that were not actively translated such as spurious mRNA or rRNA. These artifacts need to be identified and removed before or during the reference genome alignment step. The resulting clean RP data can then be used for multiple purposes including mapping of translation sites such as initiation regions or elongation regions, characterization and timing of protein folding (when combined with ChIP), and quantification of genome-wide translation via counting of ribosomal footprints per gene ^4^. The last can be utilized to assess changes in mRNA translation in individual cells or different groups of cells in response to certain drugs or therapeutics, providing insights into how such treatments work on the gene and pathway level and how these effects differ across patients or patient cohorts. In an ideal scenario, such information could then be utilized to develop predictive biomarkers to personalize treatment.

Before scientists can readily analyze RP data, key challenges must be overcome ^5^. These include the provisioning of adequate hardware and software resources to meet the data processing and storage requirements for this type of analysis. Depending on the size of the project, both can be substantial. In addition, setting up a suitable RP data processing and analysis workflow requires significant bioinformatics programming resources and careful workflow parameterization. Analysis and visualization of high-dimensional RP data is not trivial requiring a thorough understanding of multivariate data analysis and statistical methods for appropriately modeling the data ^6^. Even if all these challenges are addressed, ensuring fully reproducible results when all steps are being re-executed is very hard to accomplish unless all components are tightly integrated and automated, and software versions, arguments, and reference data are properly controlled.

Here we present RP-REP ^7^, a new software that allows scientists to address these challenges and, at the same time, facilitates full reproducibility starting from the raw data. The software is designed to run on scalable cloud resources via AWS and pre-built AMI is available atami-00b92f52d763145d3. Alternatively, users can install the software on a local Ubuntu machine using our installation script ( *RPREP/ubuntu/install-software-v2.1.0.sh*). The software also allows for joint data processing analysis of both RP and RNA-Seq data leveraging functionality of our previously published RNA-Seq software (RESEQREP) ^8^. We demonstrate the joint capabilities of our RP-REP software for a published dengue virus study that collected cytosol and ER cellular fractions of human Huh7 cells pre-infection and 6 h, 12 h, 24 h, and 40 h post-infection and performed multiple replicate RNA-Seq and RP experiments (GEO: GSE69602) ^9^.

## Methods

### Implementation

**Figure 1.**
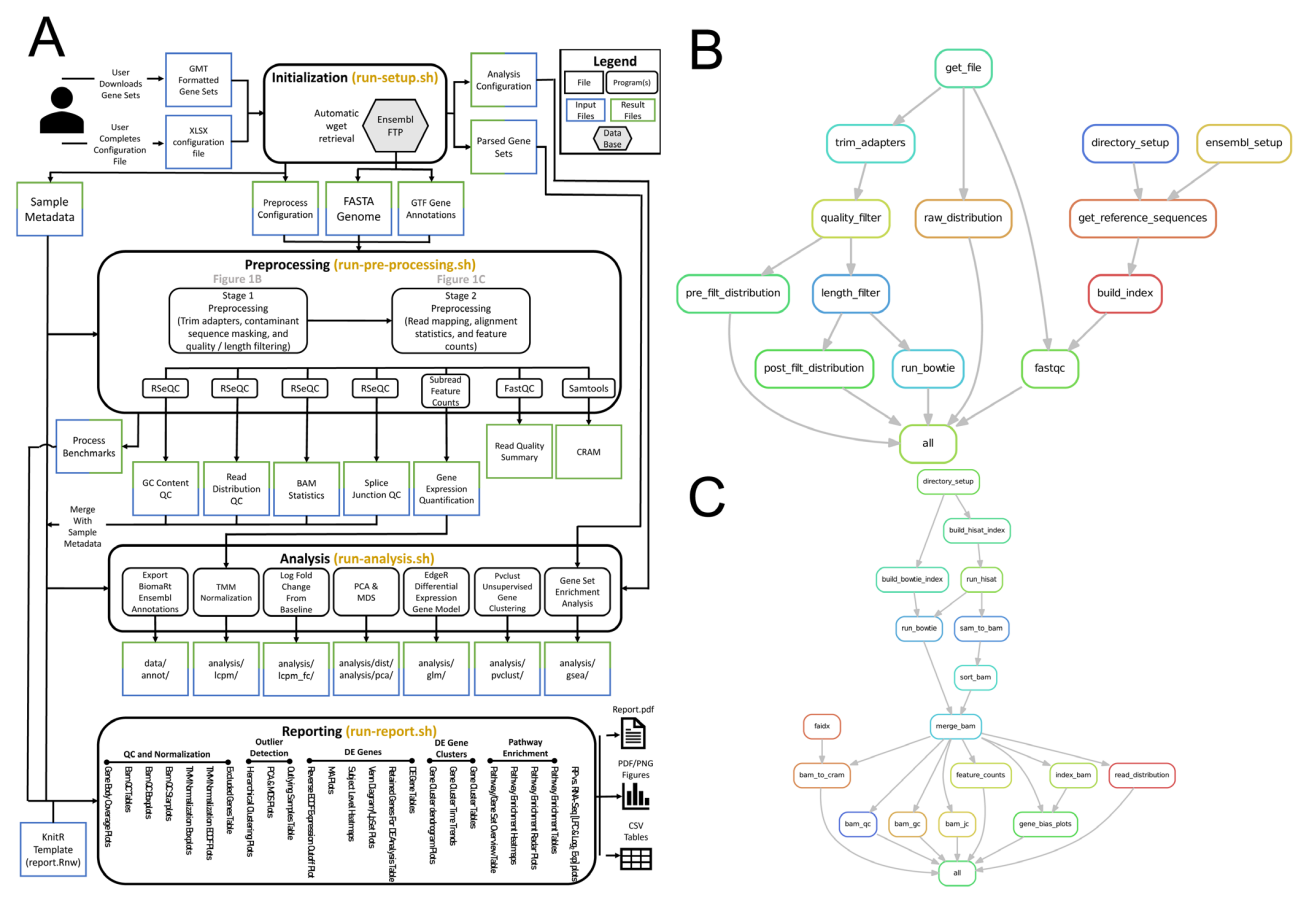
RPREP implementation overview. ( **A**) The software is organized into four main components: (1) setup (2) pre-processing (3) analysis, and (4) reporting. Preprocessing steps are organized into two stages. ( **B**) Pre-Processing Stage 1 executes read filtering steps. ( **C**) Pre-Processing Stage 2 executes read mapping and gene level quantification. ( **B**) and ( **C**) depict the respective Snakemake workflow in the form of a directed graph indicating sequential and parallel processing of certain pre-processing components.

Figure 1 provides an overview of RP-REP software components. The software is organized into four main components: (1) setup (2) pre-processing (3) analysis, and (4) reporting ( Figure 1A). The software utilizes a variety of open-source software in combination with custom shell, R, and Perl scripts to process raw sequence data, quantify gene expression, and track storage, CPU, memory, and other runtime metrics. Preprocessing steps are organized into two stages. Stage 1 executes read filtering steps ( Figure 1B) while stage 2 executes read mapping and gene level quantification ( Figure 1C).

Stage 1 performs RNA artefact filtering by retaining raw FASTQ reads that fail to map to an alignment index built from known human rRNAs, rRNA pseudogenes, tRNA pseudogenes, mitochondrial rRNAs (mt-rRNAs), mitochondrial tRNAs (mt-tRNAs), and mt-rRNA pseudogenes, as well as other known rRNA sequences from Ensembl and GenBank. Additional read processing such as adapter trimming, quality and read length filtering to retain reads that likely represent true ribosomal footprints (read length 25–35 nt), can be performed ( Figure 1B). Stage 2 performs splice-aware human reference genome alignment of reads that have been trimmed and/or filtered during Stage 1 followed by gene expression quantification carried out on the gene level, reference alignment QC including the generation of gene body read coverage plots ( Figure 1C). Processing of samples within each stage is parallelized using the Snakemake workflow management system ^10^. Dependencies of steps within each stage are outlined in Figure 1B and 1C and are optimally prioritized based on available computing resources.

The analysis component is based on R using both custom R programs as well as existing R/Bioconductor packages ( Figure 1A). The reporting component is based on R (Version 3.6.0), the knitr R package (Version 1.23), and LaTeX (Version TeX Live 2012/Debian) for reproducible and automatic PDF report and figure/table generation. All components read user-defined arguments from the respective tab in the *RPREP/config/config.xlsx* spreadsheet.

### Operation

All four workflow components can be run in sequence via the *RPREP/run-all.sh* script ^7^ or can be run individually to update results of the respective component. When running each individual step, the most recent version of the configuration file will be reloaded to ensure that any modifications to the configuration will be reflected. This is particularly useful for optimizing results by removing outliers, adjusting cut offs and for overall report customization such as color-coding.

***Step 1.*** Configuration parsing and setup: The *RPREP/run-setup.sh* script executes a parsing of the configuration .xlsx file, downloads the genome and gene models, and prepares the preprocessing and analysis/report result directories.

***Step 2A.*** Stage 1 data preprocessing: The *RPREP/source/shell/run-pre-processing.sh* script initiates the preprocessing workflow, reading in all user-specified arguments provided in the config.xlsx file. Reference data including user-specified versions of the human reference genome sequence and associated gene model information from the Ensembl database are accessed ^11^. Input for pathway enrichment analysis is handled via Gene Matrix Transposed (GMT) files. GMT files, Entrez Gene IDs, Ensembl Gene IDs, and gene symbols are supported and will be automatically mapped to the human Ensembl reference annotations. We recommend that users obtain reference pathway GMT files from the Molecular Signatures Database (MSigDB) ^12^. The MSigDB import is not automated as download requires registration, but the location of downloaded GMT file can be specified in the configuration file. We do provide a script ( *RPREP/source/shell/download-gene-sets.sh*) to automatically download Reactome, Blood Transcriptome Module, and KEGG pathway information and convert this information to GMT files (note, a license may be required prior to downloading the KEGG pathway information). Contaminant sequences of known human rRNAs, rRNA pseudogenes, tRNA pseudogenes, mitochondrial rRNAs (mt-rRNAs), mitochondrial tRNAs (mt-tRNAs), and mt-rRNA pseudogenes, as well as other known rRNA sequences from Ensembl and GenBank are downloaded using biomaRt software (Version 2.40.0) and the Ensembl Perl API (Version 90). Following the reference data download, a Bowtie2 index ^13^ of contaminant masking sequences will be created to optimize reference alignment searches. Based on FASTQ file input specifications in the config.xlsx, workflow execution downloads and decrypts (optional) FASTQ files from AWS S3 cloud storage, a local file location, or directly from SRA ^29^ via file references. Following the download, the script executes sequence data QC (FastQC). Next, 3' and 5' adapter sequences are trimmed from reads using Cutadapt (Version 2.3) ^14^. Reads with Phred quality score of less than 20 for the majority of bases are removed using FASTQ quality filter from the FASTX Toolkit software package (Version 0.0.14) ^15^. During processing of ribosomal profiling data, reads that fall outside the typical length range of ribosomal footprints (25 nt to 35 nt) are removed. Reads are then aligned to the index of contaminant sequences using Bowtie2 (Version 2.3.5) with its local alignment option. Reads that map to contaminant sequences are removed, and those that do not are output to a FASTQ file for alignment to the human reference genome. With the exception of read length filtering, the RNA-Seq data is processed as described above.

***Step 2B.*** Stage 2 data preprocessing: The human reference genome assembly, gene models, and associated gene annotation information in the form of a gene transfer format (GTF) are obtained from the ENSEMBL database. The genomic reference is built by merging all human chromosomes. Sequence reads from the Stage 1 data preprocessing that failed to map to the index of contaminant sequences are re-aligned to the reference genome using the HISAT2 splice-aware read aligner (Version 2.1.0) ^16^ on stranded, unstranded, or paired-end read data as specified in the config.xlsx, as well as reference based compression ( samtools ^17^). Ensembl gene models are used to guide the alignment process. For each sample, the quality of reference alignments is evaluated using RSeQC software (Version 3.0.0) ^18^. Gene expression quantification is carried out on the gene level using the featureCounts function as implemented in the Subread software (Version 1.6.4) ^19^. Reads that overlap with multiple genes or map to multiple genomic locations on the reference genome are excluded. This is followed by assessment of gene body coverage to calculate the average read coverage over reference genome gene sequences using the RSeQC software. Additionally, for both Stage 1 and Stage 2, the workflow will track program arguments, program return codes, input and output file names, file size, MDS checksum, wall clock time, CPU time and memory consumption using the built-in Snakemake benchmarking utility.

***Step 3.*** Data Analysis: The *RPREP/run-analysis.sh* script initializes analysis datasets for the final reporting steps including distance matrix calculations for global multivariate analysis (PCA, MDS, heatmaps), fold change calculations, and differentially expressed gene (edgeR ^20^), co-expressed gene clusters (pvclust ^21^), and enriched pathway (GoSeq ^22^) identification. Interim result files generated as part of these analyses are saved in gzipped .csv format within the analysis directory.

***Step 4.*** Automatics report generation: The *PREP/run-report.sh* script produces the final results. It runs R analyses on the intermediate analysis files generated in Step 3 and generates a summary PDF report and result tables in gzipped .csv format as well as individual figure files in .pdf and .png format. This script also summarizes key run time statistics that were collected in the Snakemake benchmarking Step 2 in graphical form.

### Minimal System Requirements

For local instance storage (storage immediately accessible by the instance’s operating system), a 60 GiB Elastic Block Store (EBS) volume is sufficient for storing the Ubuntu Linux operating system, user accounts, and temporary analysis space for smaller studies like the dengue virus case study. For studies with larger sample sizes and sequence coverage, we recommend adding one or more additional EBS volumes (see information on AWS set-up on GitHub under *RPREP/aws/aws_instructions.docx*). We found an m5.2xlarge computational Elastic Compute Cloud (EC2) instance type (8 vCPUs, 32 GiB) to be sufficient for processing and analyzing the dengue virus case study data. Our benchmarks showed that the memory-limiting step is the index generation process executed by HISAT2/Bowtie2 during the preprocessing steps. For the dengue virus case study, the maximum memory requirement was 20 GB, and we expect comparable requirements for studies of similar size.

### Installation

We provide a pre-configured RP-REP AMI available on AWS (AMI ID: RPREP RSEQREP (Ribosome Profiling and RNA-Seq Reports) v2.1 (ami-00b92f52d763145d3)) that combines the Ubuntu Linux operating system Version 18.04.2 (long- term support) with all additional software that is required for RP-REP operation ( *RPREP/software.xlxs*). We prepared a manual that provides step-by-step instructions on how to set up the AWS instance including mounting of EBS volumes for local storage and an optional Elastic IP for machine access ( *RPREP/aws/aws_instructions.docx*). Alternatively, we provide installation scripts that can be executed on a local Ubuntu machine (Version 18.04.2) to install necessary dependencies ( *RPREP/ubuntu/install-software.sh*). In both cases, (AWS or local setup) prior to workflow execution, users would need to pull the latest RP-REP source code from GitHub (git clone).

### Configuration

RP-REP configuration is handled via the *RPREP/config/config.xlsx* file. The first tab allows users to specify sample metadata. Fields include subject ID, sample ID, sampling time point, a flag (is_baseline) that indicate if a sample was collected prior to treatment, the treatment group, specimen type (e.g. B-cells, PBMCs, etc.), FASTQ sequence file location (AWS S3, local, or remote SRA location), and assay type (ribosomal_profling or rna_seq). In addition, color-coding for time points, treatment groups, and specimen types can be defined. The second tab specifies options related to the pre-processing step. This tab uses a two-column key value pair format to define options. For example, to specify the Ensembl version, users can set the value of the ensembl_version key to 95. Other options include the type of data (stranded: yes/no), paths to all software utilities, and options for executing certain workflow processes (read distribution, FastQC). Paired-end experiments can be accommodated for each sample by specifying two input FASTQ files. The third tab allows users to customize analysis and reporting components. Options include specification of cut-offs to define lowly-expressed genes, differentially expressed (DE) genes, and enriched pathways, as well as the distance metric for heatmap and gene clustering analysis. For further information, see descriptions and examples for each of these options in the configuration file ( *RPREP/config/config.xlsx*). We implemented the framework to dynamically adjust the report presentation depending on the number of subjects, time points, specimen types, and treatment group combinations. For example, Venn diagrams are shown for comparisons between up to five sets (e.g. five time points). Larger sets are accommodated via UpSet plots ^23^. The configuration file allows users to subset the data by limiting the metadata file to samples, treatment groups, and time points of interest.

### Use case

To demonstrate the functionality of the RP-REP software, we analyzed a public dengue virus (DNV) data set (GEO: GSE69602) ^9^. The study assessed the impact of DNV infection on viral and host transcription (via RNA-Seq) and translation (via RP) in human Huh7 cells after 6 h, 12 h, 24 h, and 40 h post infection. Prior to running RNA-Seq and RP, Huh7 cells were fractionated to extract RNA and ribosome-bound RNA from cytosolic and ER compartments to understand how viral replication impacts each cellular fraction on the transcriptional and translational level. The same was done for mock infected Huh7 cells to determine results for uninfected cells. DNV is a plus-strand virus; as such it depends on the host to replicate and translate itself.

Here, we used RP-REP to assess how the host transcriptional and translational profile changed over time following DNV infection. The mock-infection sample timepoint was labeled with 0h. For RNA-Seq, 2 replicates were run per time point and cellular department for a total of 20 samples. For RP, 4 replicates were run for a total of 40 samples. The results (RP-REP report) and corresponding configuration file with public SRA FASTQ file references can be found as extended data in data files 1 and 2, respectively ^7^. We provide the configuration file to exemplify the use case and to allow users to reproduce the case study analysis on their own RPREP/RSEQREP AWS instance or Ubuntu Linux machine.

The RP-REP report for this study includes 182 figures and 82 tables (data file 1 ^7^). Differential gene expression and translation was assessed by comparing pre- vs. post DNV infection read counts using negative binomial models as implemented in the edgeR R package ^20^. Genes with an FDR-adjusted p-value < 0.05 and fold change ≥4 fold were selected as differentially expression (DE) or differentially translated (DT). The high fold change cut off was chosen to accommodate the strong signal post-DNV infection which required more stringent filtering of DE/DT genes. In the following sections we highlight a subset of the key findings (referenced supplemental tables and figures refer to the corresponding results in data file 1 ^7^).

**Figure 2.**
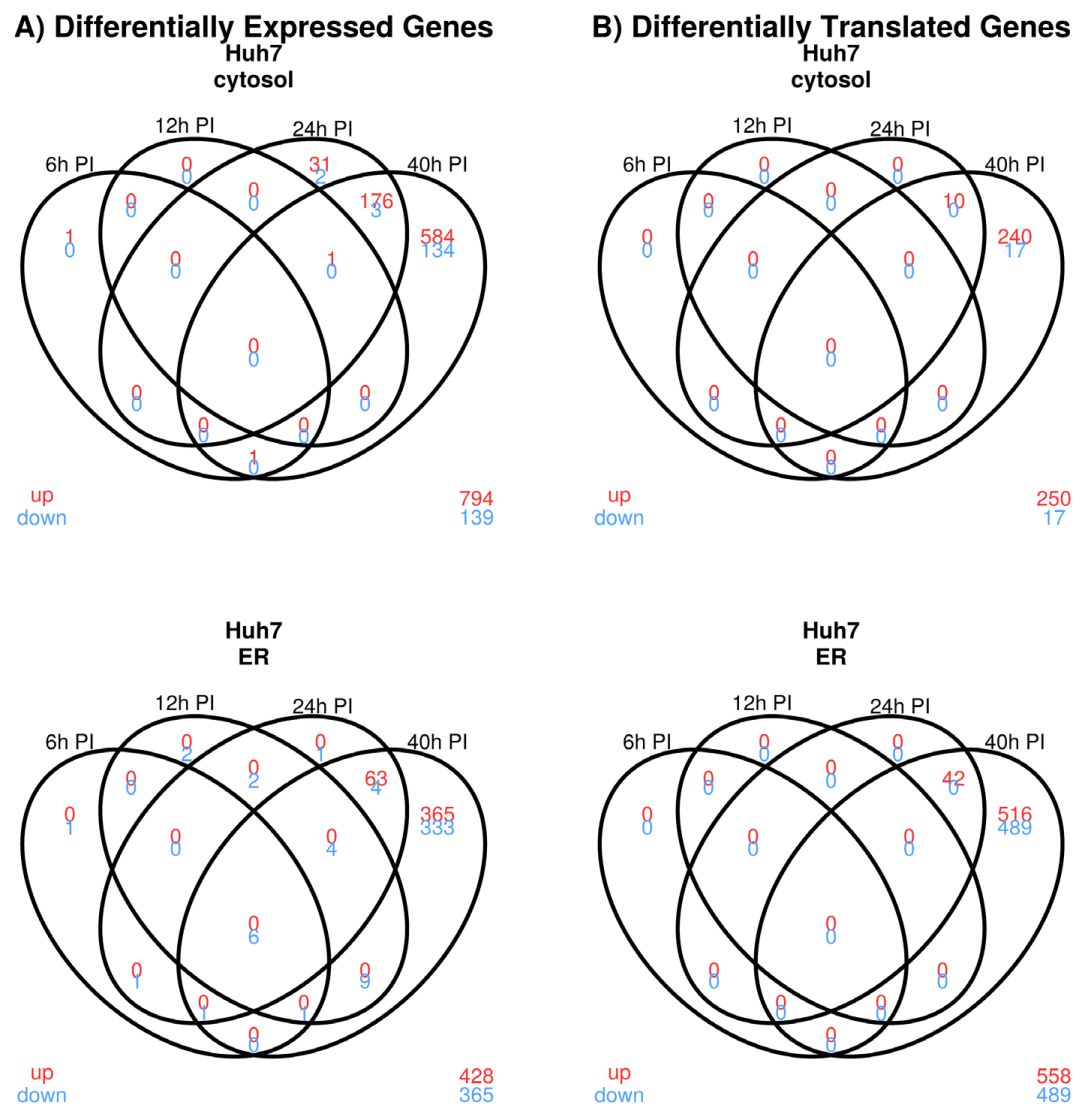
Dengue virus case study: Venn diagrams summarizing differential genes and transcripts following dengue virus (DNV) infection. In red: up-regulated relative to pre-DNV infection. In blue: up-regulated relative to pre-DNV infection. ER: endoplasmic reticulum.

**Figure 3.**
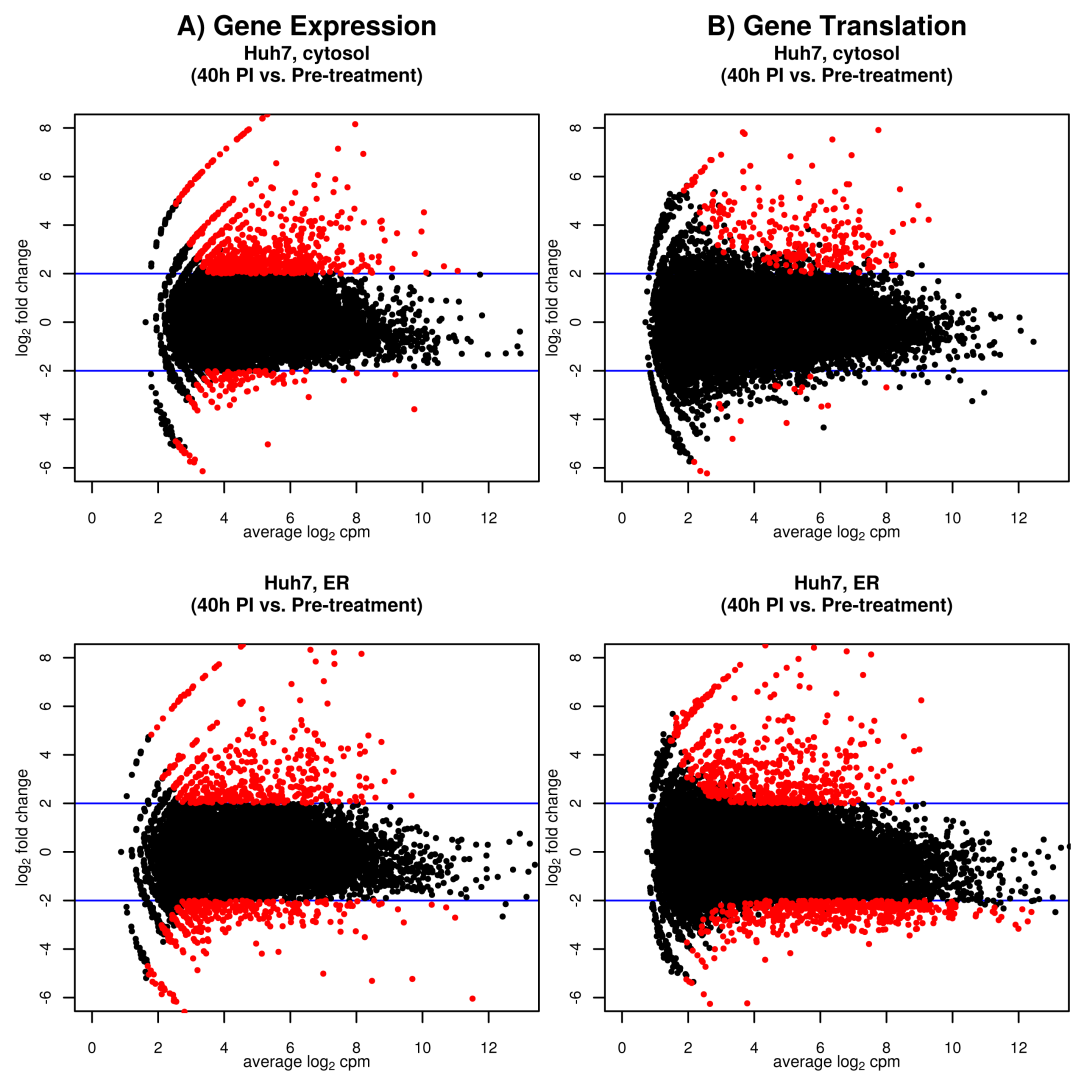
Dengue virus case study: MA plots that contrast log _2_ fold change by average gene expression and translation 40 h post infection in the cytosol and endoplasmic reticulum (ER). In red: DE/DT genes. CPM: gene expression measured in counts per million. Blue line: fold change cut off (4-fold on the original scale).

**Host gene transcription following DNV infection.** A noticeable increase in differential transcript abundance in the cytosol of infected Huh7 cells was observed at 24 h (213 DE genes) and 40 h (899 DE genes) ( Figure 2A). In the ER, DE gene expression increased from 10, to 24, to 82, and 786 DE genes at 6 h, 12 h, 24 h, and 40 h following DNV infection, respectively. While most of the DE genes expressed in the cytosol were up-regulated (98% at 24 h and 85% at 40 h), up-regulation was suppressed in the ER relative to the cytosol, in particular at 40 h (77% at 24h and 54% at 40 h) ( Figure 3A). At 24 h and 40 h, 37 (14%) and 285 (20%) DE genes overlapped between the two compartments. All DE gene results are presented in data file 1 Tables 5–12 ^7^. Pathway enrichment analysis showed that at 40h post DNV infection, transcripts in the Huh7 cytosol were enriched in GO ENDOPLASMIC RETICULUM (178 DE genes), GO IMMUNE SYSTEM PROCESS (146 DE genes), REACTOME IMMUNE SYSTEM (79 DE genes), GO RESPONSE TO ENDOPLASMIC RETICULUM STRESS (59 DE genes), REACTOME INTERFERONG SIGNALING (26 DE genes), and REACTOME UNFOLDED PROTEIN RESPONSE (25 DE genes). While similar immune system pathways were enriched in the ER compartment including GO IMMUNE SYSTEM PROCESS (111 DE genes), REACTOME IMMUNE SYSTEM (58 DE genes), and INTERFERON SIGNALING (25 DE genes), ER-related stress pathways were not enriched. All pathway enrichment results based on DE genes are provided in data file 1 Tables 19–41 ^7^.

**Host gene translation following DNV infection.** For the cytosol fraction, 24 h following infection, 10 differentially translated (DT) genes were identified ( Figure 2B). This signal increased to 267 DT genes at 40h post-DNV infection. Most of these DT responses were decreased relative to pre-infection. In the ER compartment, 42 and 1047 DT genes were detected at 24 h and 40 h post-DNV infection, respectively. The ratio of genes with increased translation was 100% for cytosol and 100% for the ER at 24h. While the ratio remained similar for cytosol at 40 h (94%), it dropped to 53% in the ER compartment indicating that protein translation in infected Huh7 cells was strongly suppressed in the ER relative to the cytosol compartment between 24 h and 40 h ( Figure 3B). The fraction of shared DT responses between compartments was 9/43 (21%) of DT genes at 24h and 192/1122 (17%) at 40 h indicating that in addition to suppression, fewer genes translated in the cytosol were translated in the ER between 24 h and 40 h. All DT gene results are presented in data file 1 Tables 46–49 ^7^.

Pathway enrichment analysis showed that at 40 h post DNV infection, translation in the Huh7 cell cytosol was enriched in GO IMMUNE SYSTEM PROCESS (58 DT genes), GO DEFENSE RESPONSE TO VIRUS (22 DT genes), GO RESPONSE TO TYPE I INTERFERON (12 DT genes), REACTOME INTERFERON SIGNALING (18 DT genes), and REACTOME CYTOKINE SIGNALING IN IMMUNE SYSTEM (22 DT genes). Most DT genes showed increased translation relative to pre-infection indicating that in the cytosol host proteins related to viral defense were actively translated. In contrast, protein translation in the ER 40 h post-DNV infection was characterized by decreased translation of host RNA related to genes involved in ER-related pathways. This included GO LIPID METABOLIC PROCESS (135 DT genes, 117 DT genes decreased), GO ENDOPLASMATIC RETICULUM (159 DT genes, 136 DT genes decreased), go intrinsic component of plasma membrane (124 DT genes, 110 DT genes decreased), GO CELL SURFACE (65 DT genes, 54 DT genes decreased), REACTOME METABOLISM OF LIPIDS AND LIPIDPROTEINS (59 DT genes, 51 DT decreased), and REACTOME POST TRANSLATIONAL PROTEIN MODFICATION (26 DT genes, 25 DT genes decreased). While some immune responses were still active at 40 h post DNV infection in the ER including REACTOME INTEREFERON SIGNALING (24 DT genes, 1 DT decreased), many immune system-related genes were deprioritized (GO IMMUNE SYSTEM PROCESS had 100 DT genes of which 49 were decreased relative to pre-infection). All pathway enrichment results based on DT genes are provided in data file 1 Tables 54–73 ^7^.

**Figure 4.**
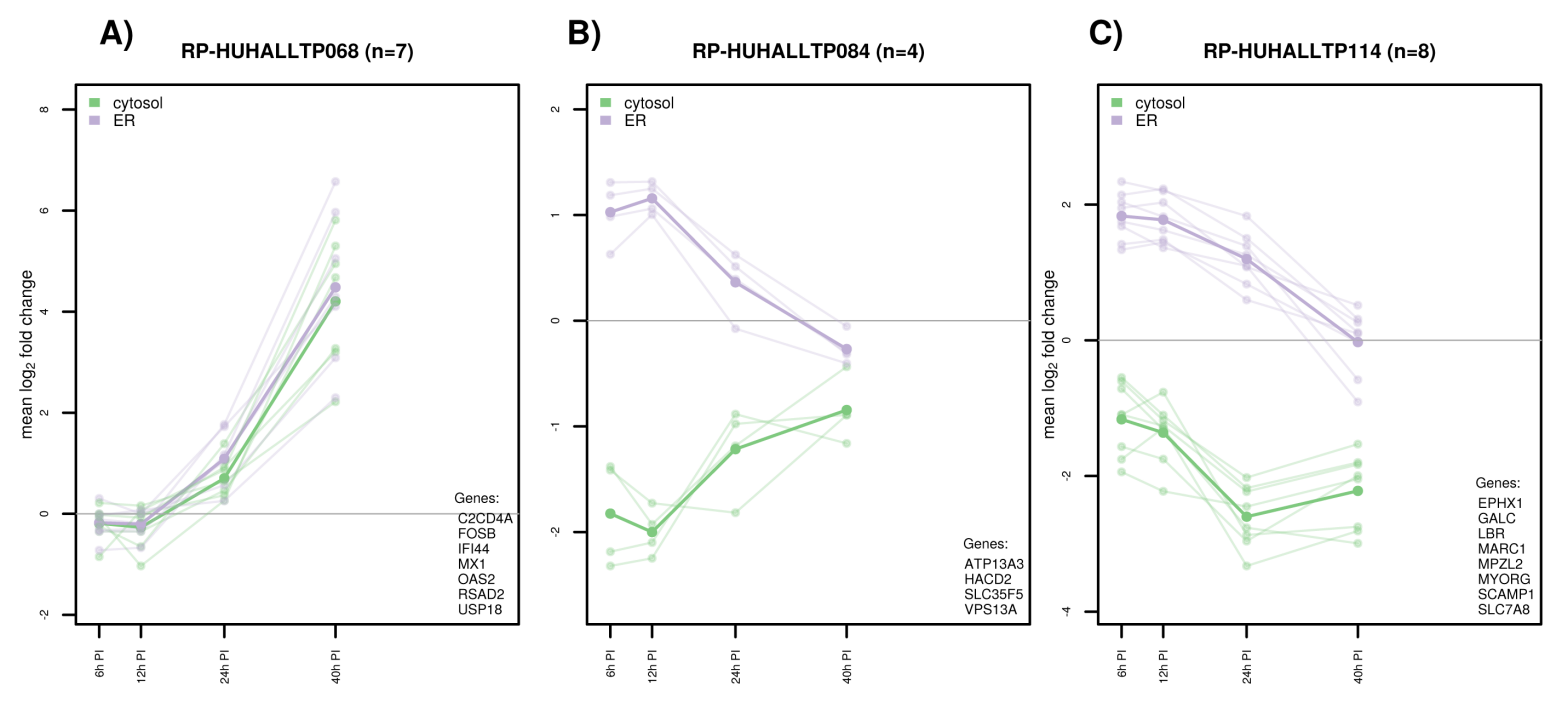
Dengue virus case study: Selection of co-translated gene cluster time trends. Each thin line represents the mean log _2_ cluster response for a certain gene. The thick line indicates the mean across genes. Co-translated gene clustered were identified using a bootstrap-based method as implemented in pvclust ^21^ using the uncentered Pearson correlation distance in combination with complete linkage clustering. ER: endoplasmic reticulum.

Time trend plots for co-translated DT genes are provided in data file 1 Figures 127–142 ^7^. A selection is shown in Figure 4. The first cluster highlights translational activation of a group of known interferon-inducible anti-viral genes ( Figure 4A). The trend line indicated that the antiviral response was first triggered between 12 h and 24 h post DNV-infection with an exponential increase in translation between 12 h and 40 h in both the cytosol and the ER. In contrast, translation of several genes encoding for proteins involved in lipid biosynthesis ( *HACD2*), lipid transfer between ER and mitochondria ( *VPS13A*), and transport ( *ATP13A*, *SLC35F5*) sharply declined between 12 h and 40 h, suggesting increased competition in the ER between viral and host translation ( Figure 4B). Translation in the cytosol for this cluster increased over time potentially to account for the loss in the ER. A similar pattern for the ER compartment was seen for a group of genes related to lipid metabolism ( Figure 4C).

## Discussion

RNA-Seq and RP are powerful sequencing-based tools to comprehensively assess cellular responses to treatment on the transcriptional and translational level, respectively. To extract meaning from such data is not trivial, requiring both computational resources as well as programming and biostatistical skills. While a multitude of RNA-Seq and RP software tools and R packages are available ^24– 26^, software that fully automate all steps starting from the raw sequencing data and ending with publication-ready tables, figures, and report are rare. Here we presented RP-REP, a new cloud-enabled software that enables researchers to analyze and contrast both RP and RNA-Seq data. The benefit of this software is that it facilitates reproducible research by automating key analysis steps including RP-specific data preprocessing including RNA contaminant filtering, reference alignment, expression/translation quantification, data QC, identification of DE/DT genes, co-expressed/translated gene clusters, and enriched pathways, and calculation of per gene translational efficiency. The software can be tailored to project needs and user data via a user-friendly configuration file. The open-source nature of the software allows for further customization.

**Figure 5.**
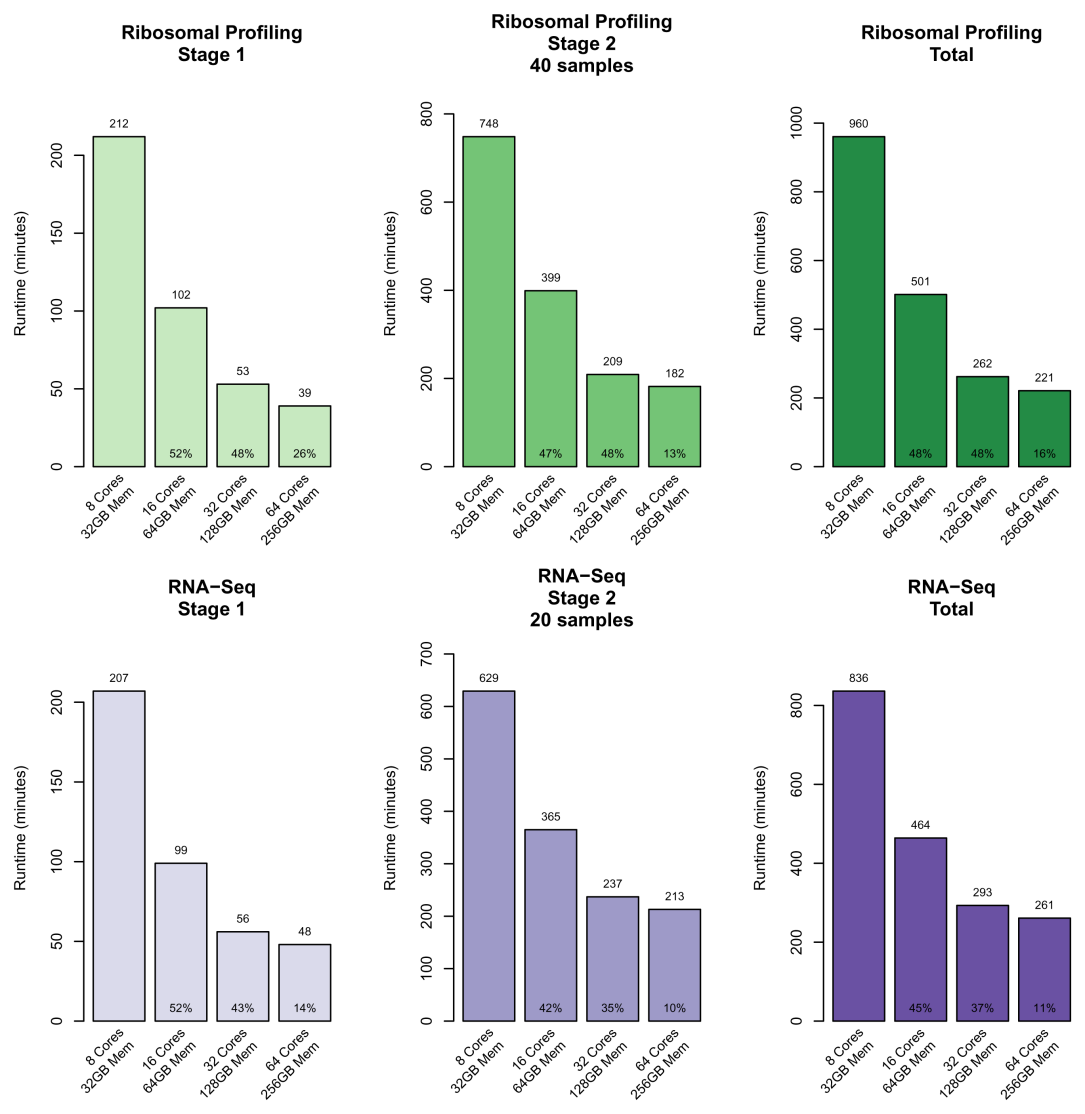
Dengue virus case study: Computational processing benchmarks for different AWS EC2 instances. The two ribosomal pre-processing stages were run using increasingly larger AWS instances to assess scalability and to estimate runtimes. The following AWS instance type were utilized: 8 Core 32GB Mem: m5.2xlarge AWS instance; 16 Core 64GB Mem: m5.4xlarge AWS instance; 32 Core 128GB Mem: m5.8xlarge AWS instance; 64 Core 258GB Mem: m5.16xlarge AWS instance.

Another benefit is that the software was designed to handle large data volumes via utilizing the Snakemake workflow system for parallel data processing. In combination with the available pre-configured AWS virtual machine image (AMI), this allows for vertical scaling of processing to 96 cores (m5.24xlarge instance, largest single instance available at the time of writing). To track computational requirements, RP-REP monitors computational metrics such as CPU and memory utilization. We used this feature to benchmark computational performance of the RP-REP preprocessing step using the dengue virus case study as an example. To evaluate performance we ran the same 60 samples on increasingly powerful but also more expensive AWS EC2 instance types: m5.2xlarge (8 vCPUs; 32 GiB RAM), m5.4xlarge (16 vCPUs; 64 GiB RAM), m5.8xlarge (32 vCPUs; 128 GiB RAM), and m5.16xlarge (64 vCPUs; 256 GiB RAM) ( Figure 5). Doubling the computational resources (CPU cores and RAM) reduced the overall runtime by about 50% when running on an m5.4xlarge compared to an m5.2xlarge and an m5.8xlarge compared to an m5.4xlarge. However, we found that the m5.8xlarge (32 vCPUs; 128 GiB RAM) machine marks the ideal convergence of processing time and cost ( Figure 5). To generate the summary PDF report for the 60 samples starting from the raw FASTQ files, sample preprocessing took around 9.25 hours on an m5.8xlarge machine (32 vCPUs; 128 GiB RAM), and analysis and reporting steps took around 9.75 hours on an m5.2xlarge machine (8 vCPUs; 32 GiB RAM). Overall, the benchmark showed that software scaled data processing well with available CPU resources.

We demonstrated the utility of RP-REP using published RNA-Seq and RP data by Reid *et al.* ^9^. Consistent with the authors findings, we found that the largest changes in transcription and translation occurred between 24 h and 40 h post DNV-infection in the cytosol and ER. Reid *et al.* and others showed that the virus hijacks a cell’s ER to prioritize viral protein synthesis over non-viral membrane proteins ^9^. Consistent with these results, we found that host translation of genes related to the ER, lipid metabolism, and components of the plasma membrane were strongly suppressed in the ER but not in the cytosol compartment at 40 h post-infection relative to pre-infection. To protect the ER from overload and avoid excess numbers of unfolded proteins, cells can activate the unfolded protein response (UPR) regulatory system ^9^. Our pathway enrichment analysis confirmed gene expression activation of the UPR in the cytosol 40 h after DNV infection. In addition, cellular anti-viral defense mechanisms related gene signatures such as those induced following interferon signaling were activated on the transcriptional and translational level in both cellular compartments at 40 h post DNV infection. While interferon signaling related genes showed an exponential increase of translation over time in both the ER and cytosol, translation for around 50 other immune system-related genes was suppressed relative to pre-infection at 40 h post DNV infection.

## Data availability

### Source data

RNA-Seq and Ribosomal Profiling data for human Hu7 cells before and 6h, 12h, 24h, 40h after dengue virus infection available from NCBI GEO with accession number GSE69602.

### Extended data

Zenodo: emmesgit/RPREP: RPREP v1.0.0. http://doi.org/10.5281/zenodo.4428861 ^7^.

This project contains the following extended data:

- Data file 1 - case-study: rprep-report-20201230.pdf (RP-REP results for DNV case study)
- Data file 2 - case-study: config-dengue.xlsx (RP-REP configuration file for the DNV case study)

## Software availability 

Source code available from: https://github.com/emmesgit/RPREP

Archived source code at time of publication: http://doi.org/10.5281/zenodo.4428861 ^7^

The cloud-ready AMI is available at AWS (AMI ID: RPREP RSEQREP (Ribosome Profiling and RNA-Seq Reports) v2.1 (ami-00b92f52d763145d3)).

License: Subject to various licenses, namely, the GNU General Public License version 3 (or later), the GNU Affero General Public License version 3 (or later), and the LaTeX Project Public License v.1.3(c).

A list of the software contained in this program, including the applicable licenses, can be accessed at https://github.com/emmesgit/RPREP/blob/master/SOFTWARE.xlsx
