# Supplementary material for: RP-REP Ribosomal Profiling Reports: an open-source cloud-enabled framework for reproducible ribosomal profiling data processing, analysis, and result reporting
Source: F1000Res. 2021 Feb 24;10:143. [Version 1] doi: 10.12688/f1000research.40668.1 (PMC9579745; doi:10.12688/f1000research.40668.1)
Supplement: Peer review history

# Peer review: Reviewer response for version 1

**DOI:** 10.5256/f1000research.43733.r150857

**Published:** 2022-10-18

**Authors:** Jordan A. Berg

**Affiliations:** 1 Altos Labs, Redwood City, CA, USA

**Major:**

- By using Docker, this is great for reproducibility, but what if improved version of a dependency is released? How easy will it be to update the pipeline?
- Are there options to modify the footprint window? For instance, some work has shown that 21 nt RPFs correspond to open A site ribosomes ( https://www.cell.com/molecular-cell/fulltext/S1097-2765(18)31063-3).
- It would be helpful to explain more why each dependency was chosen. For instance, by Bowtie2 vs STAR? Why Cutadapt vs something else?
- It would be helpful to discuss existing pipelines for ribosome profiling data and more clearly explain the rationale for creating this pipeline.

**Minor:**

- Some of the phrasing is unclear in the introduction. For instance, the authors state that “ribosomes can be fixated together” in ribosome profiling. However, I think what is meant that ribosomes can by fixated to transcripts (via cycloheximide, etc).
- I think it would be ideal to cite all dependency software within the manuscript/references for the benefit of the authors of those software.
- The output summary PDF is quite large. Is there a way to prioritize output summaries or make them easy to parse? In the case of the case study, 623 pages seems difficult to intuitively parse, especially if I were a user with minimal ribosome profiling experience. For the results, could headers/sections be specified for each analysis type so users could navigate to the results they are interested in easier?
- It would be nice to quantify how large the input FASTQ files were in the benchmarking to help users predict how long processing would take for their project based on the size of their input files. Also, how would a human dataset scale. Presumable, the sequence search space would be different for this organism, and may impact the processing time requirements.

**Software**:

- The masking sequences in the source code are for humans, but the dataset tested for this work is not. What was the rationale for doing so, and would the user need to modify masking sequences manually for their organism of interest? I may have just not been clear about this in the writing, but I think clarification would be helpful. It seems like other scripts (e.g. download-ensembl-genome.sh) are also hardcoded for humans.

Are the conclusions about the tool and its performance adequately supported by the findings presented in the article?

Yes

Is the rationale for developing the new software tool clearly explained?

Yes

Is the description of the software tool technically sound?

Yes

Are sufficient details of the code, methods and analysis (if applicable) provided to allow replication of the software development and its use by others?

Yes

Is sufficient information provided to allow interpretation of the expected output datasets and any results generated using the tool?

Yes

Reviewer Expertise:

Metabolism, ribosome profiling, software development

I confirm that I have read this submission and believe that I have an appropriate level of expertise to confirm that it is of an acceptable scientific standard.

## References

[1] : High-Resolution Ribosome Profiling Defines Discrete Ribosome Elongation States and Translational Regulation during Cellular Stress. *Molecular Cell* .2019;73(5) : 10.1016/j.molcel.2018.12.009 959-970.e5 10.1016/j.molcel.2018.12.009 PMC641104030686592

# Peer review: Reviewer response for version 1

**DOI:** 10.5256/f1000research.43733.r80139

**Published:** 2021-03-10

**Authors:** Christopher Nicchitta, Molly Hannigan

**Affiliations:** 1 Department of Cell Biology, Duke University School of Medicine, Durham, NC, USA

This manuscript introduces a high utility software/data processing package that is effectively a “soup to nuts” RPF data processing suite. Paraphrasing from the authors presentation, RP-REP works with local FASTQ files, cloud-storage files (AWS S3), and SRA files. The software includes a useful set of customizable steps that are integral to RFP data processing and analysis. It appears to be particularly strong in alignment QC, data visualization and pathway analysis, in addition to parameters such as gene-specific translational efficiency metrics. RP-REP meets a growing need and as an open source tool, this contribution supports community development and standardization of Ribo-seq computational pipelines. The authors do a commendable job of highlighting the challenges of the ribosome profiling data analysis (paragraph 2 of the Introduction) and the utility of RP-REP to the community can be expected to be high.

**Major Comments:**

 The manuscript would be strengthened by including a short paragraph in the Introduction that informs the reader of currently available resources, e.g. GWIPS-viz, riboSeqR, riboWaltz, RiboCode, and notes that there are limited resources for fully integrated analyses. In the latter category, RiboToolKit, from the Gregory lab, stands out as having similar functionality to RP-REP. In the Discussion, it would be helpful to briefly contrast RIboToolKit and RP-REP, so that the general reader can better appreciate the utility of each. In particular, it would be helpful to note that RP-REP is open source, cloud-enabled, and can run on both virtual machines and locally. RP-REP appears to be better positioned for pathway analysis, biomarker discovery, drug/therapeutic response analyses, etc., whereas RIboToolKit delves more deeply into modes of translational regulation, codon optimality, translational dynamics.

 3) The analysis pipeline does not appear to normalize RPFs (Ribo-seq) to RNA abundance (RNA-seq). Rather, it appears that it processes RNA-seq and ribosome profiling data independently. This should be clarified as such normalization is standard in Ribo-seq analyses. For example, the authors do not describe if they intersect ribosome profiling with parallel RNA-seq (in Figure 1 or within the body of text).

**Minor Comments: **

1. Abstract – clarify the statement “…by sequencing short mRNA fragments undergoing translation in ribosomes.” This should read “…sequencing ribosome-protected mRNA fragments obtained by ribonuclease digestion of polyribosomes. These fragments represent ribosome-occupied regions of the mRNA.”
2. Intro: the sentence “ *The technique makes use of the facts that mRNAs that undergo translation in ribosomes can be fixated to each other using certain chemicals and that* *the joint ribosome/mRNA complexes can be isolated using chromatography after mRNAs not protected by ribosomes have been degraded using ribonucleases*” needs rewording. In the technique, polyribosomes are isolated under native conditions and/or after addition of translation elongation inhibitors such as cycloheximide or emetine, which generally stabilize polyribosomes through processing. The mRNAs are not fixed to one another.
3. Use case. Second paragraph, fifth line – change department to compartment.
4. Data export in .svg formats would also be useful.
5. Does the analysis include read length distribution plots? These are useful for distinguishing between true RPFs and RNA binding protein-protected fragments. Similarly, does the analysis include frame periodicity output? Although the utility of this metric is not entirely clear, given that it can be influenced by the nuclease used in the RPF generation stage and whether the periodicity analysis is anchored at the 5’ or 3’ end of the read, it is useful output to include.

Are the conclusions about the tool and its performance adequately supported by the findings presented in the article?

Yes

Is the rationale for developing the new software tool clearly explained?

Yes

Is the description of the software tool technically sound?

Yes

Are sufficient details of the code, methods and analysis (if applicable) provided to allow replication of the software development and its use by others?

Yes

Is sufficient information provided to allow interpretation of the expected output datasets and any results generated using the tool?

Yes

Reviewer Expertise:

RNA localization, translational regulation, RNA-seq, Ribo-seq.

We confirm that we have read this submission and believe that we have an appropriate level of expertise to confirm that it is of an acceptable scientific standard.
